# Enquête CAP (Connaissances, aptitudes, pratiques) auprès de la population et des professionnels de santé sur la santé bucco-dentaire et le noma en 2012 dans la région du Sahel, Burkina Faso

**DOI:** 10.48327/mtsi.v5i4.2025.640

**Published:** 2025-11-17

**Authors:** Hadissa TAPSOBA, Jocelyne Valérie GARÉ, Aissata Sané CONGO, Lamoussa Robert ZOMA, Ernest Robert TOÉ

**Affiliations:** 1 Projet de sensibilisation et de renforcement des capacités pour la prévention du noma au Burkina Faso (PSRCPN/BF), Ouagadougou, Burkina Faso; 2 Réseau pour la promotion de la santé bucco-dentaire et la recherche en Afrique, Ouagadougou, Burkina Faso; 3 Département de santé publique, Unité de formation et de recherche en sciences de la santé (UFR/SDS), Université Joseph Ki Zerbo, Ouagadougou, Burkina Faso; 4 Réseau pour la promotion de la santé bucco-dentaire et la recherche en Afrique, Ouagadougou, Burkina Faso; 5 Institut national de la statistique et de la démographie-INSD, Ouagadougou, Burkina Faso (au moment de l’étude); 6 Ministère de l’économie, des finances et de la prospective, Ouagadougou, Burkina Faso; 7 Clinique dentaire ERAS, Ouagadougou, Burkina Faso

**Keywords:** Santé bucco-dentaire, Noma, Enquête CAP, Séno, Oudalan, Yagha, Soum, Burkina Faso, Sahel, Afrique subsaharienne, Oral health, Noma, KAP survey, Séno, Oudalan, Yagha, Soum, Burkina Faso, Sahel, Sub-Saharan Africa

## Abstract

**Introduction:**

Le noma est un enjeu majeur de santé publique dans la région du Sahel au Burkina Faso. Pour lutter contre ce fléau, des acteurs se sont mobilisés pour construire un projet en s’appuyant sur l’état des connaissances de la population et des professionnels sur cette pathologie.

**But de l’étude:**

Étude descriptive sur les connaissances, attitudes et pratiques relatives au noma et à la santé bucco-dentaire dans la région du Sahel en 2012.

**Résultat:**

Bien que la population ne connaisse pas le terme « noma », les symptômes en sont généralement connus. Les connaissances des causes d’altération de la santé bucco-dentaire sont plus confuses. Si la population est consciente des besoins d’hygiène dentaire et du possible rôle de l’alimentation, des efforts de sensibilisation sont nécessaires. Une faille réside dans les capacités du système de santé. Les agents de santé sont insuffisamment formés. Les tradipraticiens restent peu impliqués sur le sujet tout comme les médias qui pourraient jouer un rôle clé.

**Conclusion:**

Cette étude a eu un impact sur la mise en œuvre du programme de lutte contre le noma et son évolution notamment au regard de trois stratégies majeures: la mobilisation sociale, la stratégie communautaire, le renforcement des capacités des agents de santé.

## Abstract (en)

**Introduction:**

Noma is a significant public health concern in the Sahel region of Burkina Faso. To combat this issue, stakeholders have come together to develop a project based on the actual knowledge of local populations and medical professionals about the disease.

**Purpose of the study:**

This descriptive study examines knowledge, attitudes, and practices related to noma and oral health in the Sahel region in 2012.

**Results:**

Although the population is unfamiliar with the term “noma”, they generally know the symptoms. However, knowledge of the causes of poor oral health is more confused. While the population is aware of the need for dental hygiene and the possible role of diet, more awareness-raising efforts are needed. There is a gap in the health system’s capacity. Health workers are insufficiently trained. Traditional practitioners and the media remain uninvolved in the issue, despite their potential to play a key role.

**Conclusion:**

This study impacted the implementation and development of the noma control program, particularly with regard to three major strategies: social mobilization, community strategy, and capacity building for health workers.

## Introduction

Le noma a été défini comme un problème majeur de santé publique pour l’Organisation mondiale de la santé (OMS), en raison de sa forte létalité, des graves mutilations faciales chez les survivants et de son impact important sur la vie socio-économique des survivants et de leurs familles [11]. L’essentiel des cas survient en Afrique subsaharienne [12]. D’origine multifactorielle et de causes mal définies, le noma atteint principalement les enfants âgés de 2 à 6 ans souffrant de malnutrition, d’une mauvaise hygiène bucco-dentaire, d’un affaiblissement du système immunitaire ou d’une maladie infectieuse, et vivant dans des conditions de pauvreté [5,14]. En décembre 2023, l’OMS a officiellement intégré le noma dans la liste des maladies tropicales négligées, soulignant la nécessité de renforcer la lutte contre cette maladie [8]. Le Burkina Faso figure parmi les pays les plus touchés. La prévalence y a été estimée en 2018 à près de 100 000 cas sur 23 millions d’habitants dont seulement 10% traités [9].

**Figure 1:**
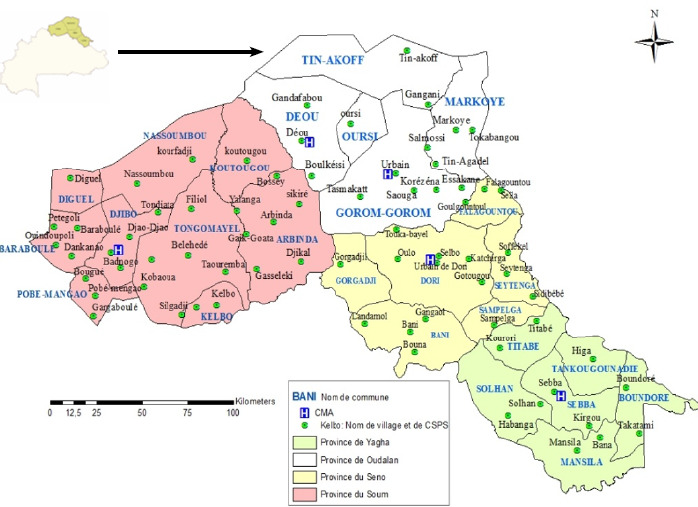
Carte sanitaire de la région du Sahel du Burkina Faso (source: BNDT 2002 (IGB), DRS/Sahel)

Au Sahel, la situation est particulièrement préoccupante en raison d’un environnement sociosanitaire déficient caractérisé par un climat rude, un faible taux de scolarisation, une insécurité alimentaire, des pesanteurs socioculturelles, une faible couverture sanitaire et une forte mobilité du personnel sanitaire (Fig. 1). La région est marquée par une forte prévalence de la malnutrition [13]. Le Projet de sensibilisation et de renforcement des capacités pour la prévention du noma au Burkina (PSRCPN/BF) s’investit dans ce domaine depuis 2011. En 2012, avant le démarrage des activités, il s’est agi de dresser un état des connaissances, attitudes et pratiques (CAP) de la population, des soignants et des tradipraticiens liées au noma et à la prévention bucco-dentaire. Le PSRCPN/BF a été conjointement mis en place par le Réseau pour la promotion de la santé bucco-dentaire et la recherche en Afrique et l’ONG allemande *Gegen Noma,* en collaboration avec le ministère de la Santé. Les principaux objectifs poursuivis sont:

- l’amélioration de la connaissance du noma, des facteurs de risques, et des mesures préventives par le grand public;
- le renforcement de la mobilisation sociale et la participation communautaire dans les activités relatives à la prévention des affections bucco-dentaires et du noma;
- le renforcement des capacités des prestataires de soins pour la prévention et la prise en charge précoce des affections bucco-dentaires et du noma;
- l’amélioration de l’accès à des soins curatifs et préventifs de qualité dans les centres de santé, en particulier pour les enfants;
- la formation des agents de santé communautaire pour la communication en faveur d’un changement de comportement et de la recherche des cas de noma;
- l’intégration de la prévention du noma et des affections bucco-dentaires dans des structures et programmes de santé existants.

La région du Sahel a fait l’objet d’une priorité pilote dans ses quatre provinces (Oudalan, Séno, Soum et Yagha) et ses quatre districts sanitaires (Djibo, Dori, Gorom-Gorom et Sebba). L’objectif de cet article est de présenter les principaux résultats de l’étude CAP réalisée avant la mise en place du projet.

## Matériels et méthodes

L’étude a porté sur l’ensemble de la région du Sahel incluant la population générale ainsi que les acteurs de santé, les agents de santé communautaire et les tradipraticiens. Une étude CAP a été réalisée [7].

Les groupes cibles visés en particulier étaient les enfants de moins de 7 ans, les enfants et adolescents de 12 à 17 ans, les personnes âgées de 18 ans et plus ayant au moins un enfant de 0 à 6 ans. Cette enquête s’est également intéressée à tous les intervenants clés du domaine de la santé. Des entretiens et/ou questionnaires ont été réalisés auprès des agents professionnels de santé, des agents de santé communautaire et des tradipraticiens (Annexe).

Le plan de sondage utilise la technique du sondage stratifié à deux degrés. Le critère de stratification est le milieu de résidence. Dans chaque province, deux strates ont été identifiées: le milieu urbain et le milieu rural. L’enquête a eu lieu dans chacune des strates. Au premier degré, la base de sondage est constituée de la liste des zones de dénombrement (ZD) établies par la cartographie censitaire du recensement général de la population et de l’habitation du Burkina Faso (en 2006). Au deuxième degré, la base de sondage est constituée de la liste des ménages dénombrés dans chaque ZD hébergeant en leur sein au moins un enfant de 0 à 6 ans. Nous avons procédé au tirage des ZD avec une probabilité proportionnelle à leur taille. Les ménages ont été tirés à probabilité égale dans chaque zone. Seuls les ménages comptant en leur sein au moins un enfant de 0 à 6 ans ont été retenus. Le chef d’équipe a tiré les 12 ménages à enquêter.

Toutes les personnes de 12 à 17 ans, de plus de 18 ans et ayant à leur charge un enfant de 0 à 6 ans ont été interrogées. Dans un ménage sur deux, tous les adultes de plus de 18 ans ont été également enquêtés.

La taille de l’échantillon de la population requise par province a été calculée pour une précision de 5% dans les proportions estimées et avec un facteur correctif pour tenir compte des non-réponses. La taille de l’échantillon répondant à ces critères est d’au moins 400 femmes, adolescents et adultes respectivement. Sur la base de 15 ZD à couvrir par province, 12 ménages ont été enquêtés par ZD. Pour les professionnels de la santé, l’échantillonnage a été exhaustif.

À défaut d’une base de sondage exhaustive des tradipraticiens et agents de santé communautaire, tout le personnel soignant travaillant dans la formation sanitaire du village et à proximité de la ZD a été enquêté. L’enquête visait à recueillir:

- le niveau des connaissances et croyances relatives à la santé bucco-dentaire, aux affections bucco-dentaires et au noma;
- les attitudes des populations vis-à-vis du noma et des affections de la cavité buccale en général;
- les comportements liés à la santé bucco-dentaire;
- la situation de base des enfants de moins de 7 ans en termes d’état de santé bucco-dentaire et de noma, et les pratiques relatives à leur alimentation et hygiène bucco-dentaire;
- les pratiques des prestataires de soins en matière de prévention et traitement des affections bucco-dentaires et du noma;
- les principales sources d’information sur la santé dans la région et les canaux de communication les plus appropriés pour la population.

Nous avons utilisé sept questionnaires, dont un par groupe cible: ménage, enfant de 0 à 6 ans, enfant et adolescent de 12 à 17 ans, adulte de 18 ans et plus, agent de santé, agent de santé communautaire, tradipraticien. Les entretiens se sont déroulés en langues locales. Douze enquêteurs ont été sélectionnés. Un pré-test a permis de finaliser les outils et d’anticiper certaines difficultés de terrain. La collecte des données sur le terrain a duré trois semaines. L’ensemble des personnes interrogées a donné son accord. Les données ont été transmises et analysées de manière anonyme. Quatre bases de données ont été constituées et transférées dans le logiciel SPSS: « Ménage », « Agent de santé », « Agent de santé à base communautaire », « Tradipraticien ».

## Résultats

Les résultats de l’enquête conduite en 2012 sont présentés en cinq grandes parties:

- la description de la population étudiée;
- les connaissances, attitudes et pratiques sur le noma, les stomatites et les affections buccodentaires (le terme « stomatite » est employé au sens large pour désigner toute inflammation de la muqueuse buccale);
- la perception et les croyances sur la santé bucco-dentaire;
- les comportements liés à la santé bucco-dentaire;
- la qualité de vie.

Seuls les éléments saillants sont présentés dans cet article.

L’étude a été menée sur un échantillon de 637 ménages. Dans cet échantillon, 417 femmes en charge d’un enfant de moins de 7 ans ont été interrogées, ainsi que 418 adolescents de 12 à 17 ans et 1 570 adultes âgés de 18 ans ou plus. L’étude a permis de recueillir des données auprès de 292 agents de santé, 272 agents de santé à base communautaire et 170 tradipraticiens.

Le nombre moyen de personnes par ménage est estimé à 5 personnes, le nombre d’enfants de moins de 7 ans entre 1 et 4. Le nombre d’adolescents âgés de 12 à 17 ans varie entre 1 et 5 par ménage. La moyenne du nombre d’adultes est de 2 par ménage. La grande majorité des ménages est dirigée par des hommes (94,3%). La région est caractérisée par un niveau d’analphabétisme très élevé (90% des chefs de ménage ne savent ni lire ni écrire).

Une proportion importante des ménages au Sahel consomme de l’eau potable selon les critères locaux (62% des ménages), les autres utilisent des puits non protégés, les rivières et les marigots. Seulement un quart des ménages (24%) utilise des latrines simples ou ventilées et 0,5% utilise une chasse d’eau. L’évacuation des ordures se fait surtout dans les tas d’immondices (36%) et la rue (29%). Les fosses à ordures sont utilisées dans 15% des cas, les bacs à ordures publics dans 5% des cas, le ramassage privé dans 1% des cas. Près de 90% des ménages possèdent des animaux à proximité du domicile. Les soins de santé se font en priorité dans les centres de santé pour 95% des ménages et auprès de tradipraticiens pour 5% d’entre eux. La grande majorité de la population n’a jamais entendu parler de noma (seuls 3,9% en a entendu parler). Cette proportion est plus élevée dans le Séno (9,4%) et plus faible dans le Yagha (1,6%). Parmi les appellations locales du noma, les principales sont: *bassewui, bassowel* et *gondé* (les deux premières sont en fulfudé, la dernière n’est pas rattachée à une langue précise). Mais 90% de la population ne connait pas l’appellation du noma dans sa langue. Cependant, les symptômes sont connus et identifiés comme suit: les maux de dents, de gencives ou de bouche (67%), les plaies dans la bouche (66%) et les gonflements de la joue (37%). Les principales sources d’information sur le noma sont les parents ou membres de la famille (41%), les amis (22%), la télévision (20%), le voisinage (20%) et la radio (17%).

Selon les agents de santé (Tableau I), le noma peut se définir par les affections bucco-dentaires (78%), les gingivites et stomatites (18%), les ulcères nécrotiques (2%) et les maladies de la peau (1%). Ils caractérisent les symptômes par l’inflammation de la joue (62%), la fièvre/douleur (28%), la déformation du visage (22%) et la mauvaise haleine (22%). Certains citent le bec de lièvre (8%). Ils identifient les signes cliniques suivants: plaie à la joue, nez et bouche rongés (43%), tuméfaction buccale (24%), haleine fétide (23%). Selon eux, plusieurs facteurs sont à l’origine du noma, les principaux étant une mauvaise hygiène buccodentaire (64%) et la malnutrition (51%). Certains citent la mauvaise haleine comme faisant partie des causes probables (10%). Pour les caractéristiques cliniques, ils citent les plaies extensives de la bouche/saignement des gencives/rougeur (51%), des douleurs ou de la fièvre (17%) et les dents visibles (15%). À noter que la perte de substance, qui est une caractéristique du noma à la phase d’état, est peu connue (seuls 8% la citent).

Plus de la moitié (56%) des agents de santé communautaires (Tableau II) a déjà entendu parler du noma (61% dans le Séno et 38% dans l’Oudalan). Parmi ceux qui en ont déjà entendu parler, la plupart (97%) définit le noma comme étant une maladie touchant la bouche et le visage. La majorité reconnaît le noma à travers les plaies dans la bouche (76%). Cependant, ils citent d’autres symptômes (diarrhée, éruption cutanée) non reconnus comme caractéristiques du noma. Pour eux, la principale cause probable du noma est la mauvaise hygiène bucco-dentaire (50%). Une bonne partie ne connaît pas les facteurs favorisant le noma (33%). Une faible proportion incrimine la sorcellerie ou la malédiction. La majorité d’entre eux connaît la localisation du noma, en l’occurrence la bouche (90%) et le visage (38%). Quelques-uns localisent le noma au niveau du ventre (4%). Les services de santé constituent la principale source d’information sur le noma (72%). La radio (14%), la télévision (7%) et la presse écrite (6%) occupent une place non négligeable dans cette information. Environ deux tiers des tradipraticiens ont déjà entendu parler du noma (80% dans le Séno et 32% dans le Yagha). Parmi ceux qui en ont déjà entendu parler, la plupart évoque la mauvaise hygiène bucco-dentaire (51%) et la malnutrition ou la pauvreté (26%) comme causes probables. Cependant, une bonne partie en ignore les causes (29%) et certains citent la sorcellerie ou la malédiction.

La moitié de la population enquêtée (51%) pense qu’on peut prévenir le noma (80% dans le Yagha, 36% dans l’Oudalan). Les moyens de prévention les plus cités sont: des consultations régulières dans les centres de santé (61%), une bonne hygiène bucco-dentaire (51%) et une alimentation équilibrée (30%). Bon nombre des enquêtés (15%) évoque la consultation chez les marabouts (le terme « marabout » est utilisé pour désigner un tradipraticien à connotation religieuse s’appuyant sur le Coran). Les résultats sont identiques chez les femmes en charge d’un enfant de moins de 7 ans et chez les adultes de 18 ans et plus.

Une très forte proportion d’agents de santé (95%) pense qu’on peut prévenir le noma. L’hygiène buccale est le plus fréquemment citée (70%), le suivi en consultation ou le diagnostic précoce (30%), la sensibilisation à aller dans les centres de santé (29%) et la prise en charge de la malnutrition (27%). Les agents de santé s’informent sur le noma à travers la littérature médicale (36%), la télévision (29%) et les collègues (18%). Les séminaires de formation sont peu cités (7%).

Plus de la moitié des agents de santé communautaires (58%) pense qu’on peut prévenir le noma (Oudalan 80%; Séno 48%). Parmi ceux qui pensent qu’on peut le prévenir, la majorité (86%) évoque la bonne hygiène bucco-dentaire, suivie de la lutte contre la malnutrition (9%).

Parmi la population, 80% des enquêtés pensent qu’on peut mourir du noma, avec des variations entre le Yagha (100%) et le Soum (66%). Il y a peu de différence entre les sous-groupes de population. La majorité des agents de santé à base communautaire (84%) le pense également. Contrairement aux perceptions de la population, c’est dans le Yagha que l’on note une proportion en dessous de la moyenne régionale (58%).

Près de 60% des enquêtés pensent qu’une personne en bonne santé peut « attraper » le noma (71% chez les femmes ayant en charge un enfant de moins de 7 ans). Cette perception est plus prononcée dans le Yagha avec 83% (100% des femmes) et moins dans l’Oudalan avec 20% (33% des femmes). Le noma est perçu comme une maladie contagieuse par 35% de la population (50% dans le Yagha et 14% dans le Soum). Parmi les individus susceptibles d’attraper le noma, 69% des agents de santé à base communautaire ciblent les enfants (90% dans le Soum, 40% dans l’Oudalan). Une partie cite « toute personne » comme cible potentielle du noma (26%).

Parmi la population, 80% disent savoir que le noma se soigne (90% dans le Yagha, 57% dans l’Oudalan). Pour les attitudes à adopter, les interrogés suggèrent en premier lieu la visite au centre de santé et chez le tradipraticien en second lieu. Les résultats diffèrent peu entre les femmes ayant en charge un enfant de moins de 7 ans et les autres adultes. La consultation chez le marabout, le prêtre ou le pasteur et l’automédication sont faiblement citées par les femmes. À noter que dans le Séno, 60% des femmes préconisent la consultation chez le tradipraticien et 40% chez le marabout ou le pasteur ou le prêtre.

D’une manière générale, 85% des agents de santé à base communautaire pensent qu’on peut soigner le noma. La majorité préconise la consultation au centre de santé en premier lieu (96%) et en second lieu chez les tradipraticiens (8%).

Il y a une forte acceptation des malades du noma par la population générale (presque 81%) avec de faibles disparités selon les provinces. Elle est moins importante chez les femmes (65%). Les adolescents acceptent de collaborer avec un malade du noma, avec une forte disparité entre les provinces (100% d’adolescents dans le Soum et le Yagha, 0% dans l’Oudalan).

Plusieurs causes ont été évoquées pour expliquer la survenue des affections bucco-dentaires: une mauvaise hygiène bucco-dentaire (47%), la consommation du sucre (40%) et la malnutrition (19%). Plusieurs moyens sont cités pour les prévenir: brosser régulièrement les dents de l’enfant (45%), consulter régulièrement un agent de santé (44%) et éviter la consommation du sucre (39%). Il faut noter qu’une partie de la population cite comme moyen de prévention l’éviction du lait. La majorité des agents de santé (76%) déclare avoir déjà entendu parler des stomatites. Plusieurs types de stomatites sont cités: les gingivites (47%), les glossites (18%) et les parodontites (10%). Certains évoquent les caries dentaires comme un type de stomatite. Selon les agents, plusieurs traitements sont disponibles: antibiothérapie (53%), bain de bouche avec l’eau tiède salée (38%), médicament anti-inflammatoire (35%).

Seulement 18% des agents de santé mènent des activités de sensibilisation et de formation sur la santé bucco-dentaire. Seulement 2% ont été formés sur ces affections. Même constat pour les agents de santé communautaire, excepté au Séno où l’on en rencontre quelques-uns (4%). Cependant, 32% d’agents de santé communautaire ont bénéficié d’une sensibilisation sur la santé bucco-dentaire.

Un peu plus de la moitié de la population (57%) accorde aux maladies bucco-dentaires une importance supérieure aux autres problèmes de santé. De manière générale, 88% de la population est préoccupée par la santé bucco-dentaire. Plus de la moitié des enquêtés (58%) pense qu’il est important de conserver ses dents naturelles, 35% ne partageant pas cet avis.

La plupart des enquêtés (83%) partage l’opinion selon laquelle les caries dentaires donnent un mauvais aspect du visage, 96% perçoivent le danger que peuvent constituer les problèmes dentaires. L’impact des problèmes dentaires sur les autres problèmes de santé est reconnu par la grande majorité (93%). Parmi les personnes concernées par les problèmes dentaires, environ 31% ont exprimé un besoin de traitement ou d’information en particulier des conseils sur le brossage des dents (25%). Chez les femmes ayant en charge un enfant de moins de 7 ans et chez les adultes, les besoins en traitement liés à la santé bucco-dentaire sont identiques. Les adolescents ont exprimé des attentes en termes d’obturation, d’extraction dentaire et de redressement de dents à hauteur de 52% chacun.

La plupart des enquêtés (94%) perçoit l’importance de se brosser les dents. La moitié (53%) ne sait pas que l’utilisation du fil dentaire n’empêche pas la survenue des maladies des gencives. La majorité (55%) pense que les aliments sucrés provoquent la survenue de caries dentaires tandis que 34% ne partagent pas cet avis. À noter que 12% n’ont aucune connaissance sur l’action du sucre dans la survenue de caries.

Deux personnes sur trois ne savent pas que la consommation d’eau fluorée aide à la prévention des caries. Parmi ceux qui connaissent son utilité, 19% partagent cette opinion et 13% la réfutent. Pour 81% des enquêtés, le fait d’aller voir un dentiste n’épargne pas les problèmes de dents, de gencives ou de dentier. Plusieurs raisons sont évoquées pour ne pas aller chez un dentiste dont les plus importantes sont l’inexistence de problèmes dentaires (73%) et le manque de moyens financiers (18%). Parmi ceux qui sont allés consulter un dentiste, les raisons de consultation sont: un problème dentaire (50%), un besoin d’examen ou de détartrage (38%) et les consultations obligatoires (13%). Il faut noter que ces motifs de consultation ont été évoqués par les populations des provinces disposant de cabinets dentaires (le Séno et le Soum). Parmi les motifs de satisfaction évoqués par ceux ayant consulté un dentiste, les plus importants sont l’obtention d’un rendez-vous, l’accueil à la réception et l’accueil de l’assistant dentaire. La durée du trajet pour aller chez le dentiste et le quartier où se trouve le dentiste ont été relativement bien appréciés par les patients. Les motifs de satisfaction les plus importants sont l’information donnée sur le traitement, la modernité des équipements de soins et la propreté du lieu.

Des conseils sont aussi donnés par les tradipraticiens: se brosser les dents (74%), aller au centre de santé (24%), éviter de manger des nourritures très dures (6%) et éviter de consommer du sucre (21%). La population du Sahel consomme habituellement des aliments à base de céréales (66%) et des aliments sucrés (35%). Les fruits sont faiblement consommés, en lien avec l’environnement qui en est dépourvu. En dehors de la consommation habituelle de céréales (77%), la consommation du sucre est importante chez les adolescents (51%). Parmi ces derniers, 11% disent fumer du tabac et 8% chiquer.

Une part importante de la population (76%) se nettoie la bouche et les dents au moins une fois par jour. Cependant, une partie non négligeable (4%) ne se lave jamais les dents, notamment dans le Séno (12%). Le même comportement est observé chez les femmes en charge d’un enfant et les autres adultes. Chez les adolescents, le nettoyage est plus fréquent (86%) mais dans le Séno, 20% ne se brossent jamais les dents. Parmi ceux qui se nettoient la bouche et les dents, un quart (26%) utilise une brosse à dents. La plus forte proportion provient du Séno (36%) et la plus faible est observée dans le Yagha (12%). La majorité de ceux qui se brossent les dents n’utilise pas de dentifrice (82%). D’autres moyens sont utilisés dont les plus courants sont les cure-dents en bois (63%), les bâtonnets frotte-dents (52%) et le charbon de bois (9%).

Plusieurs traitements sont proposés par les agents de santé aux patients pour la prise en charge du noma: l’antibiothérapie (52%), la chirurgie (48%), les bains de bouche antiseptiques (26%). Cependant, peu d’entre eux ont déclaré avoir déjà soigné un cas de noma (12%, avec 27% dans l’Oudalan et seulement 3% dans le Soum).

Les traitements proposés aux patients souffrant de noma par les tradipraticiens sont des produits à base de plantes (95%) et à base de parties d’animaux. Plusieurs « remèdes » sont utilisés par les tradipraticiens dont les plus cités sont le *sylmidi* (34%), *l’heidi* (23%), le *sigini* (21%), le *lansongne* (19%), le *loukouhi* (16%) et le *founafouna* (11%). A noter que ces remèdes et les plantes qui les composent ne sont pas connus.

Des agents de santé disent référer les cas de noma dès le diagnostic (52%), d’autres seulement à un stade avancé (16%). Il faut cependant noter qu’une proportion importante d’agents de santé n’a jamais référé de cas. Les principaux lieux de référence sont le centre médical avec antenne chirurgicale (75%), le chirurgien-dentiste (42%) ou le centre hospitalier régional (18%). Ils réfèrent également les cas de stomatites et d’affections bucco-dentaires, dès le diagnostic (33%) ou seulement au stade avancé (31%). Beaucoup n’ont jamais référé de cas (36%). Les tradipraticiens prennent aussi en charge les affections bucco-dentaires. Il s’agit des extractions de dents (2%) et les « remplacements de dents » (1%).

La majeure partie des enquêtés (97%) n’est pas gênée de sourire ni de converser en public malgré l’apparence de leurs dents, contre 3% qui évitent à certains moments de sourire. Une bonne partie est satisfaite de l’apparence de ses dents (75%), 22% la jugent passablement satisfaisante et seulement 3% ne la trouvent pas bonne. Les trois quarts (78%) des enquêtés sont satisfaits de la santé de leurs dents et gencives, 21% la trouvent passable, tandis qu’à peine 1% l’estime mauvaise. Une grande majorité (91%) n’a pas connu de douleur ou de gène au cours des 12 derniers mois et 95% déclarent pouvoir mâcher. Certains ont relaté avoir eu des problèmes dentaires au cours des 12 derniers mois: des douleurs au chaud ou au froid (12%), des douleurs ou des saignements des gencives (11%), des saignements fréquents au brossage (8%) ou des dents qui bougent (8%). La majorité n’a pas relevé de troubles du sommeil à cause de problèmes dentaires au cours des 12 derniers mois (95%).

## Discussion

Cette enquête CAP relative au noma et à la santé bucco-dentaire dans la région du Sahel burkinabé a été réalisée en 2012 dans le cadre du Projet de sensibilisation et de renforcement des capacités pour la prévention du noma au Burkina Faso. Elle est la première étude documentant ce sujet dans cette région. Les résultats ont mis en évidence un certain nombre d’enseignements ayant permis de renseigner et d’orienter le projet. Celui-ci se poursuit en 2025 bien qu’il ait dû s’adapter à de nombreuses contraintes. La région du Sahel fait face à une grande insécurité, liée au terrorisme, qui a entraîné le départ d’une grande partie des personnels de santé et de nombreux habitants. La poursuite des activités de prévention du noma dans cette zone est devenue extrêmement difficile. En 2012, la population du Sahel connaissait très mal le noma en tant que maladie. Le terme « noma » lui-même était quasi inconnu, même si ses manifestations étaient souvent reconnues. Le noma était perçu à travers ses symptômes, mais pas identifié en tant qu’entité nosologique distincte. Il est possible que bien des répondants assimilaient en fait les cas de noma à d’autres affections orales sévères sans les distinguer. Cette probable confusion entre le noma et d’autres lésions bucco-dentaires graves a aussi été observée chez certains soignants. Cela constitue une limite dans l’interprétation des niveaux de connaissance. Nos résultats rejoignent globalement ceux observés dans d’autres études en Afrique de l’Ouest. Au Mali, une enquête auprès de tradipraticiens a montré qu’ils connaissaient empiriquement le noma avec des explications étiologiques très diverses, souvent non biomédicales [2]. Au Burkina Faso, une étude menée auprès d’agents de santé primaires dans une autre région a retrouvé des lacunes importantes dans la connaissance du noma et de sa prise en charge [3]. Nos données diffèrent de celles rapportées en Zambie rurale, où une majorité de soignants était capable d’identifier correctement le noma [1]. Ces comparaisons montrent que la connaissance du noma peut varier considérablement selon le contexte géographique et la catégorie de population, soulignant l’importance d’adapter les stratégies de formation et de sensibilisation. Les conceptions étiologiques du noma parmi la population et les tradipraticiens combinent des facteurs biomédicaux (malnutrition, hygiène) et des facteurs socioculturels ou mystiques (sorcellerie, châtiment divin). Cette coexistence d’attributions causales rationnelles et magico-religieuses a déjà été rapportée [4]. Elle rappelle l’importance d’impliquer non seulement le secteur de la santé mais aussi les leaders communautaires et religieux dans la lutte contre le noma, afin de prendre en compte les croyances pouvant nuire aux parcours de soins.

Cependant, si la manière de contracter le noma reste parfois floue (avec des confusions entre causes et symptômes), ou erronée (contagiosité), la population et les soignants identifient les principaux moyens pour l’éviter notamment en termes d’hygiène. Ceci est d’autant plus remarquable qu’ils vivent dans des conditions d’hygiène très précaires. Les pratiques déclarées face au noma suggèrent un comportement généralement approprié (recours aux structures de santé) avec une persistance notable de la confiance envers les tradipraticiens. Qu’une moitié environ de la population envisage de consulter un guérisseur traditionnel en cas de noma reflète la réalité d’un système de santé pluraliste au Sahel, où médecine moderne et approche traditionnelle coexistent. Il est intéressant de noter que ce recours au tradipraticien est mentionné par de nombreuses femmes, y compris en complément d’une consultation au centre de santé: l’approche de la maladie par les tradipraticiens est essentiellement tournée vers la cure de la cause mystique identifiée, généralement par divination. Plutôt que d’opposer ces recours, il paraît pertinent de favoriser une complémentarité encadrée, par exemple en intégrant les tradipraticiens dans les campagnes de dépistage et d’orientation des cas de noma. L’intérêt de leur intégration au système de santé a déjà été souligné dans différents contextes [8]. Ceci semble d’autant plus pertinent qu’en termes de soins, les agents de santé, les agents de santé communautaire et les tradipraticiens diffèrent peu dans leurs pratiques. Cependant, ces acteurs sont globalement insuffisamment formés pour accompagner la population, tant pour prévenir, repérer ou orienter. Cela vaut pour le noma mais également pour les autres affections dentaires. Ce constat est similaire à ceux qui ont pu être faits au Burkina Faso ou dans d’autres pays d’Afrique [1,2, 3]. Il rend d’autant plus prioritaire le volet « Renforcement des capacités des prestataires de soins et des agents de santé communautaire dans la région du Sahel ».

En termes de santé bucco-dentaire, la population observe un comportement relativement protecteur en reconnaissant l’intérêt du brossage dentaire et les dangers d’une alimentation sucrée. La santé dentaire est perçue comme un sujet relativement important. Des efforts de sensibilisation sont à faire en ce qui concerne l’usage d’eau fluorée, de dentifrice ou encore le recours préventif aux soins dentaires. Pour les recours aux soins, la population s’oriente en majorité vers les centres de santé. Ces éléments représentent un atout important sur lequel s’appuyer. La région Afrique a connu la plus forte progression mondiale des maladies buccodentaires ces dernières décennies, témoignant du faible accès à la prévention et aux soins [10]. À signaler cependant que si l’état bucco-dentaire est un déterminant de noma, le rôle spécifique de l’hygiène bucco-dentaire dans la prévention du noma reste peu documenté à ce jour [6].

Pour le second volet intitulé « Mobilisation sociale et sensibilisation dans la région du Sahel », il convient de noter l’importance non négligeable des médias dans la sensibilisation de la population. Ces médias semblent mieux fonctionner que les séances de formation/sensibilisation qui pourraient être proposées.

Les données recueillies ont apporté un éclairage sur les progrès à apporter dans la région du Sahel. Elles ont permis d’identifier les actions à privilégier, celles où le changement doit s’opérer (notamment dans la couverture de toutes les mesures préventives) et la nécessité de mieux les structurer. Ceci a eu un impact sur la conduite du projet. Celui-ci a réalisé une série d’activités de communication, y compris une caravane de presse, une campagne de diffusion radiophonique, des rencontres de plaidoyer ainsi que des représentations théâtrales. Il a permis la constitution et l’animation de groupes d’apprentissage et de suivi des pratiques d’alimentation du nourrisson et du jeune enfant.

Plusieurs points doivent être soulignés. Il s’agit d’une étude déclarative, basée sur des entretiens. Les réponses obtenues peuvent être influencées par un biais de désirabilité sociale, les participants cherchant à donner la réponse qu’ils estiment attendue. Ce biais est classique dans les enquêtes CAP. Nous avons tenté de le minimiser par la formation des enquêteurs. Il n’est pas exclu que certaines connaissances ou bonnes pratiques aient été surestimées.

La collecte ayant eu lieu en 2012, les données reflètent la situation d’il y a plus de 10 ans. Entretemps, le contexte régional a profondément changé, en particulier à partir de 2015 avec la crise sécuritaire. En 2025, la région du Sahel est confrontée à une insécurité extrême liée au terrorisme, qui a entraîné le départ de la plupart des agents de santé et des enseignants de la zone. Les populations civiles et les forces armées subissent des attaques régulières. Ce contexte rend la mise en œuvre des activités de prévention et de prise en charge du noma extrêmement difficile. Les actions se poursuivent mais de manière limitée. Malgré ces limites, notre enquête a permis d’orienter les actions du programme PSRCPN/BF dès 2013. Face aux lacunes constatées, des modules de formation spécifiques sur le noma ont été intégrés pour les agents de santé. Des supports de sensibilisation communautaire ont été élaborés. Les tradipraticiens volontaires ont été intégrés dans le dispositif de signalement des cas suspects et incités à collaborer avec les centres de santé. Des données fragmentaires suggèrent une amélioration de la connaissance du noma dans la région au fil des années du programme (communication personnelle, PSRCPN/BF). En outre, les autorités sanitaires ont inscrit la lutte contre le noma à leur agenda. L’OMS a officiellement reconnu le noma comme une maladie tropicale négligée (MTN) en 2023. Cette reconnaissance internationale devrait contribuer à mobiliser davantage de ressources et d’efforts pour sa surveillance, sa prévention et sa prise en charge [15]. Néanmoins, les données épidémiologiques sur le noma restent insuffisantes pour estimer son poids réel [5]. Des initiatives globales sont désormais nécessaires pour combler ce manque d’information, notamment *via* l’intégration du noma dans les systèmes de santé et de collecte de données des pays endémiques, conformément aux recommandations de l’OMS sur les MTN [11]. Avec toutes les limites des études descriptives, l’étude CAP réalisée pourrait être reproduite afin de caractériser une tendance d’impact du projet.

## Conclusion

L’enquête conduite en 2012 dans le cadre du Projet de sensibilisation et de renforcement des capacités pour la prévention du noma au Burkina Faso est la première enquête d’ampleur conduite dans ce pays, et notamment au Sahel, sur une maladie non transmissible, le noma, et plus globalement la santé bucco-dentaire. Elle a mis en lumière un important déficit de sensibilisation et de formation, mais aussi l’existence de relais communautaires pouvant être mobilisés. Malgré un contexte socio-sanitaire et sécuritaire difficile, les enseignements de cette enquête restent d’actualité pour guider les stratégies de lutte contre le noma.

## Remerciements

Nous remercions toutes les personnes, organisations et institutions qui ont rendu ce travail possible. Toute notre gratitude aux autorités administratives et sanitaires de la région du Sahel pour leur implication et la facilitation du déroulement de l’étude. Nous remercions tout particulièrement pour leur contribution les acteurs de terrain qui ont joué un rôle déterminant dans la collecte des données et toutes les personnes qui ont accepté de répondre à nos questions en dépit de leurs multiples occupations. Nous sommes extrêmement reconnaissants à Mr Jean-Jacques Santarelli, président de l’ONG *Gegen Noma,* ainsi qu’à tous les partenaires techniques et financiers du PSRCPN/ BF au Burkina Faso, en Allemagne et en France.

## Autorisation administrative, consentement

Les données utilisées pour cet article l’ont été dans le cadre d’un diagnostic de santé publique préparatoire au projet. Ce diagnostic a été conduit conformément aux directives du ministère de la Santé du Burkina Faso. Les protocoles d’intervention et les procédures de collecte des données de suivi et d’évaluation ont été approuvés par le ministère. La collecte de données n’a été effectuée qu’après avoir obtenu le consentement éclairé verbal préalable de chaque personne participant à un questionnaire, avec une information claire fournie, incluant le droit de refus. La confidentialité a été strictement respectée, notamment par l’anonymisation de tous les documents avant leur transmission.

## Financement

Gegen Noma (Allemagne), Vaincre Noma (France), Ministère fédéral allemand de la Coopération économique et du développement.

## Contributions des auteurs et autrices

Hadissa TAPSOBA a rédigé la première version de l’article. L’ensemble des auteurs et autrices a été impliqué dans la conception de l’étude et dans l’analyse des données et a relu, contribué à la finalisation de l’article et approuvé la version finale.

## Déclaration de liens d’intérêt

Aucun lien d’intérêt n’a été déclaré.
