# Supplementary material for: KAP’ (Knowledge Attitude Practices) survey of the population and health professionals regarding oral health and noma in 2012 in the Sahel region, Burkina Faso
Source: Med Trop Sante Int. 2025 Nov 17;5(4):mtsi.v5i4.2025.640. [Article in French] doi: 10.48327/mtsi.v5i4.2025.640 (PMC12948366; doi:10.48327/mtsi.v5i4.2025.640)
Supplement: Related texts

# Research article: KAP’ (Knowledge Attitude Practices) survey of the population and health professionals regarding oral health and noma in 2012 in the Sahel region, Burkina Faso

## Introduction

The World Health Organization (WHO) has defined noma as a major public health problem due to its high case fatality rate, the severe facial mutilation suffered by survivors, and its significant impact on the socioeconomic lives of survivors and their families [11]. Most cases occur in sub-Saharan Africa [12]. It has multiple causes and poorly defined origins and mainly affects malnourished children aged two to six with poor oral hygiene, weakened immune systems, and infectious diseases and living in poverty [5,14]. In December 2023, the WHO officially added noma to the list of neglected tropical diseases, highlighting the need to increase efforts to combat the disease [8]. Burkina Faso is one of the countries most affected. In 2018, the prevalence was estimated at nearly 100,000 cases among 23 million inhabitants, only 10% of which were treated [9]. The situation in the Sahel is particularly concerning due to its poor socio-sanitary environment, which is characterized by a harsh climate, low school enrollment rates, food insecurity, sociocultural constraints, poor health coverage, and the high mobility of health personnel (Fig. 1). The region has a high prevalence of malnutrition [13]. Since 2011, the Project for Awareness Raising and Capacity Building for the Prevention of Noma in Burkina Faso (PSRCPN/BF) has worked in this area. Before the start of activities in 2012, the project aimed to assess the knowledge, attitudes, and practices (KAP) of the population, of the healthcare providers, and of traditional practitioners regarding noma and oral health prevention. PSRCPN/BF was established jointly by the Network for the Promotion of Oral Health and Research in Africa and the German NGO *Gegen Noma,* in collaboration with the Ministry of Health. The main objectives were to:

- improve the general public’s knowledge of noma, its risk factors, and preventive measures.
- strengthen social mobilization and community participation in activities related to preventing oral diseases and noma;
- strengthen the capacity of healthcare providers to prevent and manage oral diseases and noma early on;
- improve access to quality curative and preventive care in health centers, particularly for children;
- training community health workers in communication for behavior change and noma case finding;
- training community health workers in communication for behavior change and noma case finding;
- integrate noma and oral health disease prevention into existing health structures and programs.

The Sahel region, specifically its four provinces (Oudalan, Séno, Soum, and Yagha) and four health districts (Djibo, Dori, Gorom-Gorom, and Sebba), was the focus of a pilot priority.

This article presents the main results of the KAP study conducted prior to the project’s implementation.

## Materials and methods

The study covered the entire Sahel region and included the general population, health professionals, community health workers, and traditional practitioners. A KAP study was conducted [7]. The specific target groups were children under seven, children and adolescents aged twelve to seventeen, and adults aged eighteen and over with at least one child aged 0 to 6. The survey also focused on all key stakeholders in the health sector. Interviews and/or questionnaires were conducted with professional and community health workers, as well as traditional practitioners (Appendix). The survey used a two-stage, stratified sampling technique. The stratification criterion was the place of residence. In each province, two strata were identified: urban and rural. The survey was conducted in each stratum. At the first level, the sampling frame consisted of a list of census areas established through the general population and housing census mapping in Burkina Faso in 2006. At the second stage, the sampling frame consisted of a list of households with at least one child aged 0 to 6 years in each area.

We selected the areas with a probability proportional to their size. Households were selected with an equal probability in each zone. Only households with at least one child aged 0 to 6 were selected. The team leader selected the 12 households to be surveyed. People aged 12 to 17, over 18, and those with a child aged 0 to 6 in their care were interviewed. In every other household, all adults over 18 were surveyed as well.

The required sample size per province was calculated to achieve an accuracy of 5% in the estimated proportions, with a correction factor accounting for non-responses. A sample size of at least 400 women, adolescents, and adults met these criteria. Based on 15 areas to be covered per province, 12 households were surveyed per zone. For the health professionals, sampling was exhaustive. Due to the lack of an exhaustive sampling frame for the traditional practitioners and community health workers, all health personnel working in the health facility of the selected zone and in the vicinity were surveyed.

The survey aimed to collect the following information:

- The level of knowledge and beliefs related to oral health, oral diseases, and noma.
- The population’s attitudes towards noma and oral diseases in general.
- The behaviors related to oral health.
- The baseline situation of children under seven years of age in terms of oral health and noma, as well as practices related to their diet and oral hygiene.
- The practices of healthcare providers in the prevention and treatment of oral diseases and noma.
- The main sources of health information in the region and the most appropriate communication channels for the population.

Seven questionnaires were used, one for each target group: households, children aged 0 to 6, children and adolescents aged 12 to 17, adults aged 18 and over, health workers, community health workers, and traditional practitioners. The interviews were conducted in local languages. Twelve interviewers were selected. A pretest was conducted to finalize the tools and anticipate difficulties in the field. Data collection in the field took three weeks. All respondents gave their consent. The data were transmitted and analyzed anonymously.

Four databases were created and transferred to the SPSS software program: “Household,” “Health worker,” “Community health worker,” and “Traditional practitioner.”

**Figure 1:** Health map of the Sahel region of Burkina Faso (Source : BNDT 2002 (IGB), DRS/Sahel)

## Results

The results of the 2012 survey are presented in five main sections:

- Description of the study population
- Knowledge, attitudes, and practices regarding noma, stomatitis, and oral diseases. The term “stomatitis” is used broadly to refer to any inflammation of the oral mucosa.
- Perceptions and beliefs about oral health.
- Behaviors related to oral health.
- Quality of life

This article only presents the salient points.

The study was conducted using a sample of 637 households. Of those, 417 women caring for a child under the age of seven, 418 adolescents aged twelve to seventeen, and 1,570 adults aged eighteen or older were questioned. Data was collected from 292 health workers, 272 community-based health workers, and 170 traditional practitioners. On average, there were 5 people per household, with between 1 and 4 children under the age of 7 and between 1 and 5 adolescents aged 12 to 17. The average number of adults per household was two. The vast majority of households (94.3%) were headed by men. The region was characterized by a high level of illiteracy; 90% of heads of households did not read or write.

A significant proportion of households in the Sahel (62%) consumed drinking water according to local standards, while the rest used unprotected wells, rivers, and backwaters. Only 24% of households used simple or ventilated latrines, and 0.5% used flush toilets. Garbage was primarily disposed of in rubbish piles (36%) or on the street (29%). Garbage pits were used in 15% of cases, public garbage bins in 5%, and private collection in 1%. Nearly 90% of households keeped animals near their homes. Healthcare was primarily provided in health centers (95%) and by traditional practitioners (5%).

The vast majority of the population had never heard of noma; only 3.9% had. This proportion was higher in Séno (9.4%) and lower in Yagha (1.6%). The main local names for noma were *basséwui, bassowel,* and *gondé.* The first two were in Fulfulde, and the last was not linked to a specific language. However, 90% of the population did not know the name for noma in their language. Nevertheless, they knew and could identify the symptoms: toothache, gumache, or mouthache (67%); mouth sores (66%); and swelling of the cheeks (37%). The main sources of information about noma were parents or family members (41%), friends (22%), television (20%), neighbors (20%), and the radio (17%).

According to health workers, noma could be defined by oral diseases (78%), gingivitis and stomatitis (18%), necrotic ulcers (2%), and skin diseases (1%) (Table I). The symptoms were characterized as inflammation of the cheek (62%), fever/pain (28%), facial deformation (22%), and bad breath (22%). Some mentioned cleft lip and palate (8%). The following clinical signs were identified: sores on the cheeks, nose, and mouth (43%), oral swelling (24%), and foul breath (23%). They stated that several factors cause noma, the main ones being poor oral hygiene (64%) and malnutrition (51%). Some cited bad breath as a probable cause (10%). In terms of clinical characteristics, they cited extensive mouth sores, bleeding gums, and redness (51%); pain or fever (17%); and visible teeth (15%). Loss of substance, a characteristic of advanced noma, was little known (reported by only 8%). More than half (56%) of community health workers had heard of noma (61% in Séno and 38% in Oudalan) (Table II). Among those who had heard of noma, most (97%) defined it as a disease that affects the mouth and face. Most recognized noma by the sores in the mouth (76%). However, they cited symptoms such as diarrhea and skin rash that are not recognized as characteristic of noma. They believed the main probable cause of noma is poor oral hygiene (50%). Many were unaware of the factors that promote noma (33%). A small proportion blamed witchcraft or curses. Most of them knew where noma occurs: in the mouth (90%) and on the face (38%). A few located noma in the stomach (4%). Health services were the main source of information on noma (72%). Radio (14%), television (7%), and print media (6%) also played a significant role in disseminating this information.

Approximately two-thirds of traditional practitioners had heard of noma, with 80% in Séno and 32% in Yagha being familiar with it. Of those who had heard of it, most cited poor oral hygiene (51%) and malnutrition or poverty (26%) as probable causes. However, a significant proportion were unaware of the causes (29%), with some attributing it to witchcraft or curses.

Half of the surveyed population (51%) believed that noma can be prevented (80% in Yagha and 36% in Oudalan). The most commonly cited means of prevention were: regular visits to health centers (61%), good oral hygiene (51%), and a balanced diet (30%). A significant number of respondents (15%) mentioned consulting marabouts (traditional practitioners with religious connotations who rely on the Quran). These results were consistent among women caring for children under seven and adults aged eighteen and over. A very high proportion of health workers (95%) believed noma can be prevented. The most frequently cited preventive measure is oral hygiene (70%), followed by regular check-ups or early diagnosis (30%), raising awareness about health center visits (29%), and treating malnutrition (27%). Health workers learned about noma through medical literature (36%), television (29%), and colleagues (18%). Training seminars were rarely cited (7%).

Over half of community health workers (58%) believed noma can be prevented (Oudalan: 80%; Séno: 48%). Among those who believed noma can be prevented, the majority (86%) mentioned good oral hygiene, followed by combating malnutrition (9%).

Eighty percent of the population believed that noma can be fatal, with variations between Yagha (100%) and Soum (66%). There was little difference between population subgroups. The majority of community-based health workers (84%) also believed this. Contrary to popular belief, the proportion was below the regional average in Yagha (58%).

Almost 60% of respondents believed that a healthy person can “catch” noma, including 71% of women with children under seven years of age. This perception was more prevalent in Yagha (83%, 100% of women) than in Oudalan (20%, 33% of women). Thirty-five percent of the population perceived noma as contagious (50% in Yagha and 14% in Soum).

Of those susceptible to noma, 69% of community health workers targeted children (90% in Soum and 40% in Oudalan). Some cited “anyone” as a potential target of noma (26%).

Eighty percent of the population said they knew noma can be treated (90% in Yagha and 57% in Oudalan). When asked about the appropriate course of action, respondents suggested first visiting a health center and, second, consulting a traditional practitioner. There was little difference in the results between women with children under the age of seven and other adults. Self-medication and consultation with a marabout, priest, or pastor were rarely mentioned by women. Notably, in Séno, 60% of women recommended consulting a traditional practitioner, while 40% recommended consulting a marabout, pastor, or priest.

Overall, 85% of community-based health workers believed noma could be treated. The majority recommended first visiting a health center (96%) and then consulting a traditional healer (8%). The general population accepted noma patients at a high level (almost 81%), though there were slight disparities between provinces. Acceptance was less significant among women (65%). Adolescents were more likely to agree to collaborate with a noma patient, though there was a significant disparity between provinces (100% in Soum and Yagha, and 0% in Oudalan).

Several causes were cited to explain the occurrence of oral diseases, including poor oral hygiene (47%), sugar consumption (40%), and malnutrition (19%). Several preventive measures were mentioned: regularly brushing children’s teeth (45%), regularly consulting a health worker (44%), and avoiding sugar consumption (39%). It should be noted that some in the population cited avoiding milk as a means of prevention.

The majority of health workers (76%) said they have heard of stomatitis. Several types of stomatitis were mentioned: gingivitis (47%), glossitis (18%), and periodontitis (10%). Some mentioned dental caries as a type of stomatitis. According to the health workers, several treatments were available: antibiotic therapy (53%), rinsing with warm salt water (38%), and anti-inflammatory medication (35%).

Only 18% of healthcare workers engaged in activities that raise awareness and provided training on oral health. Just 2% had received training on these conditions. The same was true for community health workers, except in Séno, where a small percentage (4%) had received training. However, 32% of community health workers had received oral health awareness training.

Just over half of the population (57%) considered oral diseases to be more important than other health problems. Overall, 88% of the population was concerned about oral health. Of those surveyed, more than half (58%) believed it is important to keep their natural teeth, while 35% did not share this view.

Most respondents (83%) agreed that tooth decay makes the face look unattractive, and 96% recognized the potential dangers of dental problems. The vast majority (93%) recognized the impact of dental problems on other health issues. Of those affected by dental problems, about 31% expressed a need for treatment or information, especially advice on brushing their teeth (25%). The need for oral health treatment was the same among women with children under the age of seven and among adults. Fifty-two percent of teenagers expressed a desire for fillings, tooth extraction, and teeth straightening.

Most respondents (94%) recognized the importance of brushing their teeth. However, half (53%) did not know that flossing does not prevent gum disease. The majority (55%) believed that sugary foods cause tooth decay, while 34% disagreed. It should be noted that 12% were unaware of the role of sugar in tooth decay.

Only one out of three people knew that drinking fluoridated water helps prevent tooth decay. Of those who were aware of its benefits, 19% agree and 13% disagree. Eighty-one percent of those surveyed believed that going to the dentist does not prevent problems with teeth, gums, or dentures. The most common reasons for not going to the dentist were the absence of dental problems (73%) and lack of financial means (18%). Among those who had visited a dentist, the reasons for doing so were: having a dental problem (50%), needing an examination or scaling (38%), and mandatory consultations (13%). It should be noted that these reasons were cited by populations in provinces with dental practices (Séno and Soum). The most important reasons for satisfaction cited by those who had consulted a dentist were obtaining an appointment, the reception at the front desk, and the welcome from the dental assistant. Patients relatively appreciated the travel time to the dentist and the neighborhood where the dentist is located. The most important reasons for satisfaction were the information provided about the treatment, the modernity of the care equipment, and the cleanliness of the premises.

Traditional practitioners also offered advice: brush your teeth (74%), go to the health center (24%), avoid eating hard foods (6%), and avoid consuming sugar (21%).

The Sahel population usually consumed cere-al-based foods (66%) and sugary foods (35%). Fruit consumption was low due to a lack of fruit in the environment. Apart from the usual consumption of cereals (77%), sugar consumption was high among adolescents (51%). Among them, 11% reported smoking tobacco, and 8% reported chewing it.

A significant proportion of the population (76%) cleaned their mouths and teeth at least once a day. However, a significant proportion (4%) never brushed their teeth, particularly in Séno (12%). The same behavior was observed among women with children and other adults. Among adolescents, cleaning was more frequent (86%), though in Séno, 20% never brush their teeth. Of those who cleaned their mouths and teeth, 26% used a toothbrush. The highest proportion was in Séno (36%), and the lowest was in Yagha (12%). Most of those who brushed their teeth did not use toothpaste (82%). Other methods were used; the most common were wooden toothpicks (63%), tooth sticks (52%), and charcoal (9%).

Health workers offered patients several treatments for noma, including antibiotic therapy (52%), surgery (48%), and antiseptic mouthwashes (26%). However, few reported ever having treated a case of noma (12% overall, 27% in Oudalan, and 3% in Soum).

Traditional practitioners offered patients suffering from noma plant-based (95%) and animal-based products. The most commonly cited remedies are *sylmidi* (34%), *heidi* (23%), *sigini* (21%), *lansongne* (19%), *loukouh’i* (16%), and *founafouna* (11%). It should be noted that these remedies and the plants they contain are not well-known.

Some health workers said they refer cases of noma as soon as they were diagnosed (52%), while others only refered them at an advanced stage (16%). However, a significant proportion of health workers had never referred a case. The main referral locations were the medical center with a surgical unit (75%), the dental surgeon (42%), and the regional hospital (18%). Health workers also refered cases of stomatitis and oral diseases as soon as they were diagnosed (33%) or only at an advanced stage (31%). Many had never referred a case (36%). Traditional practitioners also treated oral diseases. These included tooth extractions (2%) and “tooth replacements” (1%). Most of those surveyed (97%) were not embarrassed to smile or talk in public, regardless of how their teeth look. Only 3% avoided smiling at certain times. A large proportion (75%) were satisfied with the appearance of their teeth, 22% considered it fairly satisfactory, and only 3% did not consider it good. Three-quarters (78%) of respondents were satisfied with the health of their teeth and gums. Twenty-one percent considered it fair and just 1% considered it poor. A large majority (91%) had not experienced any pain or discomfort in the last 12 months, and 95% said they could chew properly. Some reported having dental problems in the past 12 months, including pain when eating hot or cold foods (12%), gum pain or bleeding (11%), frequent bleeding when brushing (8%), and loose teeth (8%). The majority did not report any sleep disturbances due to dental problems in the past 12 months (95%).

## Discussion

This KAP survey on noma and oral health in the Sahel region of Burkina Faso was conducted in 2012 as part of the Awareness and Capacity Building Project for the Prevention of Noma in Burkina Faso. This is the first study to document this subject in the region. The results revealed several key insights that informed and guided the project. Although it has had to adapt to numerous constraints, the project is continuing in 2025. The Sahel region faces significant insecurity due to terrorism, resulting in the departure of many health workers and residents. Consequently, continuing noma prevention activities in this area has become extremely difficult.

In 2012, the population of the Sahel region knew very little about noma. Even though its manifestations were often recognized, the term “noma” itself was virtually unknown. Noma was perceived through its symptoms but not identified as a distinct disease. It is possible that many respondents equated cases of noma with other severe oral conditions, failing to distinguish between them. This probable confusion between noma and other serious oral lesions was also observed among some caregivers. This constitutes a limitation in interpreting knowledge levels. Our results are consistent with those of other studies in West Africa. In Mali, for example, a survey of traditional practitioners revealed their empirical knowledge of noma, which was accompanied by diverse etiological explanations, often non-bio-medical [2]. In Burkina Faso, a study of primary healthcare workers in a different region revealed significant knowledge gaps regarding noma and its management [3]. However, our data differ from those reported in rural Zambia, where the majority of caregivers could correctly identify noma [1]. These comparisons demonstrate that noma knowledge can vary considerably depending on the geographical context and population group. This highlights the importance of adapting training and awareness strategies. The etiological conceptions of noma among the population and traditional practitioners combine biomedical factors, such as malnutrition and poor hygiene, and sociocultural or mystical factors, such as witchcraft and divine punishment. The coexistence of rational and magical-religious causal attributions has been reported previously [4]. This underscores the importance of engaging not only the health sector, but also community and religious leaders in the fight against noma to address beliefs that could impede care pathways.

However, although noma is sometimes perceived in unclear or erroneous ways (e.g., confusion between causes and symptoms or contagiousness), the population and caregivers identify the main ways to avoid it, particularly in terms of hygiene. This is all the more remarkable given their precarious hygienic conditions.

The practices reported in response to noma generally suggest appropriate behavior, such as using health facilities, though there is a notable persistence of trust in traditional healers. The fact that approximately half of the population considers consulting a traditional healer in the event of noma reflects the reality of a pluralistic healthcare system in the Sahel, where modern medicine and traditional practices coexist. Interestingly, many women mention this recourse to traditional healers as a complement to a consultation at the health center. The traditional practitioners’ approach to illness focuses essentially on curing the identified mystical cause, usually through divination. Rather than opposing these approaches, it seems appropriate to promote structured complementarity. One way to do so is by integrating traditional practitioners into screening and referral campaigns for noma cases. The benefits of integrating traditional practitioners into the healthcare system have already been highlighted in various contexts [8]. This is particularly relevant given that healthcare workers, community health workers, and traditional practitioners differ little in their care practices. However, these professionals are generally inadequately trained to support the population in terms of prevention, detection, or referral. This applies to both noma and other dental conditions. Similar observations have been made in Burkina Faso and other African countries [1,2,3]. Therefore, strengthening the capacities of healthcare providers and community health workers in the Sahel region is of the utmost importance.

In terms of oral health, the population exhibits relatively protective behavior. They recognize the importance of brushing their teeth and the dangers of a sugary diet. Dental health is perceived as an important issue. However, awareness-raising efforts are needed regarding the use of fluoridated water, toothpaste, and preventive dental care. The majority of the population turns to health centers when seeking care. These factors represent important assets on which to build. The African region has experienced the highest global increase in oral diseases in recent decades, reflecting poor access to prevention and care [10]. However, it should be noted that, while oral health is a determinant of noma, the specific role of oral hygiene in preventing it remains poorly documented to date [6].

Regarding the second component, “Social Mobilization and Awareness-Raising in the Sahel Region,” the significant role of the media in raising public awareness is important to note. The media appear to be more effective than training and awareness sessions.

The collected data sheds light on the progress that needs to be made in the Sahel region. The data made it possible to identify which actions to prioritize, where change must take place (particularly in coverage of all preventive measures), and the need for better organization. These findings have impacted the project’s execution. The project has carried out a series of communication activities, including a press caravan, a radio broadcast campaign, advocacy meetings, and theatrical performances. These activities have enabled the formation and facilitation of learning groups, as well as the monitoring of infant and young child feeding practices.

Several points should be emphasized. First, this is a declarative study based on interviews. The responses obtained may be influenced by social desirability bias, whereby participants seek to provide the answer they believe is expected. This bias is common in KAP surveys. We attempted to minimize this bias by training the interviewers. It is possible that certain knowledge or good practices were overestimated.

Since the data was collected in 2012, it reflects a situation from over 10 years ago. In the meantime, the regional context has changed profoundly, particularly since 2015 due to the security crisis. Currently, the Sahel region is facing extreme insecurity linked to terrorism, which has led to the departure of most health workers and teachers from the area. Both civilian populations and armed forces are subject to regular attacks. This context makes implementing noma prevention and management activities extremely difficult. Activities are ongoing, albeit on a limited basis. Despite these limitations, our survey has guided the PSRCPN/BF program’s actions since 2013. In response to the identified gaps, specific noma training modules have been developed for health workers. Community awareness materials have also been developed. Volunteer traditional practitioners have been incorporated into the system to report suspected cases and have been encouraged to collaborate with health centers.

According to personal communication from PSRCPN/BF, fragmentary data suggest that knowledge of noma in the region has improved over the years of the program. Additionally, health authorities have prioritized combating noma. In 2023, the WHO officially recognized noma as a neglected tropical disease (NTD). This international recognition should help mobilize more resources and efforts for noma surveillance, prevention, and management [15]. However, epidemiological data on noma is insufficient to estimate its true burden [5]. Comprehensive initiatives are needed to address this information gap, particularly by integrating noma into the health and data collection systems of endemic countries in accordance with WHO recommendations on NTDs [11]. Despite the limitations of descriptive studies, the KAP study could be replicated to characterize the project’s impact.

## Conclusion

The survey conducted in 2012 as part of the Awareness and Capacity Building Project for the Prevention of Noma in Burkina Faso is the first large-scale survey conducted in this country, and particularly in the Sahel, on a non-communicable disease, noma, and more generally on oral health. It highlighted a significant lack of awareness and training, but also the existence of community relays that could be mobilized. Despite a difficult socio-health and security context, the lessons learned from this survey remain relevant in guiding strategies to combat noma.

## Acknowledgments

We would like to thank all the individuals, organizations, and institutions that made this work possible. We are deeply grateful to the administrative and health authorities in the Sahel region for their involvement and for facilitating the study. We would particularly like to thank the field workers who played a key role in data collection and all those who agreed to answer our questions despite their busy schedules. We are extremely grateful to Mr. Jean-Jacques Santarelli, president of the NGO *Gegen Noma,* as well as to all the technical and financial partners of the PSRCPN/BF in Burkina Faso, Germany, and France.

## Administrative authorization, consent

The data used for this article were collected as part of a public health assessment conducted in preparation for the project. This assessment was carried out in accordance with the guidelines of the Burkina Faso Ministry of Health. The intervention protocols and procedures for collecting monitoring and evaluation data were approved by the ministry. Data collection was only carried out after obtaining the prior verbal informed consent of each person participating in a questionnaire, with clear information provided, including the right to refuse. Confidentiality was strictly respected, in particular by anonymizing all documents before transmission.

## Funding

Gegen Noma (Germany), Vaincre Noma (France), German Federal Ministry for Economic Cooperation and Development.

## Authors’ contributions

Hadissa TAPSOBA wrote the first draft of the article. All authors were involved in the study design and data analysis, and reviewed, contributed to the finalization of the article, and approved the final version.

## Conflict of interest

The authors declare no conflict of interest.
